# Quality of Life in Patients with Pancreatic Cancer—A Literature Review

**DOI:** 10.3390/ijerph20064895

**Published:** 2023-03-10

**Authors:** Elżbieta Cipora, Aleksandra Czerw, Olga Partyka, Monika Pajewska, Anna Badowska-Kozakiewicz, Marta Fudalej, Katarzyna Sygit, Mateusz Kaczmarski, Edyta Krzych-Fałta, Anna Jurczak, Katarzyna Karakiewicz-Krawczyk, Sylwia Wieder-Huszla, Tomasz Banaś, Ewa Bandurska, Weronika Ciećko, Dariusz Artur Kosior, Piotr Kułak, Andrzej Deptała

**Affiliations:** 1 Medical Institute, Jan Grodek State University in Sanok, 38-500 Sanok, Poland; 2 Department of Economic and System Analyses, National Institute of Public Health NIH—National Research Institute, 00-791 Warsaw, Poland; 3 Department of Health Economics and Medical Law, Faculty of Health Sciences, Medical University of Warsaw, 01-445 Warsaw, Poland; 4 Department of Oncology Propaedeutics, Medical University of Warsaw, 01-445 Warsaw, Poland; 5 Faculty of Health Sciences, Calisia University, 62-800 Kalisz, Poland; 6 Department of Basic Nursing, Faculty of Health Sciences, Medical University of Warsaw, 01-445 Warsaw, Poland; 7 Department of Clinical Nursing, Pomeranian Medical University in Szczecin, 71-210 Szczecin, Poland; 8 Department of Radiotherapy, Maria Sklodowska-Curie Institute-Oncology Centre, 31-115 Cracow, Poland; 9 Center for Competence Development, Integrated Care and e-Health, Medical University of Gdansk, 80-204 Gdansk, Poland; 10 Mossakowski Medical Research Institute, Polish Academy of Science, 02-106 Warsaw, Poland; 11 Department of Cardiology and Hypertension with Electrophysiological Lab, Central Research Hospital, The Ministry of the Interior and Administration, 02-507 Warsaw, Poland

**Keywords:** pancreatic cancer, quality of life, pain management, mental adjustment to disease, literature review

## Abstract

Pancreatic cancer is the malignant disease with the highest mortality rate, and it ranks third in the world after lung and colon cancer. Identified factors that increase the risk of developing pancreatic cancer include chronic pancreatitis, radiation therapy to the pancreatic area due to another cancer, diabetes mellitus, obesity, smoking, and age. The objective of this study was to present the current state of knowledge on the quality of life of patients diagnosed with pancreatic cancer, factors that determine QoL, and ways of coping with the disease. The low curability and low survival rates of pancreatic cancer significantly affect the quality of life of patients, often in the form of significant deterioration, especially in terms of mental changes, cognitive functions, and coping with the disease. Cognitive decline with comorbid depression is also typical for patients with this type of cancer. Research has shown that the health-related quality of life of patients with pancreatic cancer is low, so further research is needed to improve the situation in this area.

## 1. Introduction

Literature data on risk factors for pancreatic cancer are limited, only in 40% of cases can these factors be identified. Nonmodifiable (hereditary) factors include primarily HBOC syndrome (hereditary breast and ovarian cancer syndrome responsible for 17–19% of cases of hereditary pancreatic cancer), HNPCC syndrome (hereditary nonpolyposis colorectal cancer), or FAMMM syndrome (familial atypical multiple mole melanoma), and age [1,2,3,4]. The development of sporadic pancreatic cancer occurs relatively often in smokers and people consuming excessive amounts of alcohol, as well as in patients with chronic pancreatitis, obesity, and most likely diabetes, as well as in people after the radiation therapy to the epigastric and abdominal area due to other cancers [5,6].

Pancreatic cancer (PC) (more than 85% of diagnoses are pancreatic ductal adenocarcinoma—PDAC) is the seventh major cause of death among neoplasms in the world [7,8,9,10]. Symptoms of pancreatic cancer are nonspecific, including unspecified abdominal pain, nausea, weight loss, and lack of appetite. Often, the diagnosis is made accidentally while performing diagnostic tests for another reason, and therefore pancreatic cancer remains one of the most lethal cancers—it ranks 3rd in the world behind lung cancer and colorectal cancer [11,12]. Body changes that may occur as a result of illness, such as weight loss, affect the quality of life. Most research in this area concerns breast cancer, where according to the results, the perception of one’s own body has a significant impact on QoL [13]. In a study by Nayak et al. 85% of the surveyed people with cancer were not satisfied with their body image [14]. Moningi et al., in a study on the subject of the correlation between clinical stage and performance status with QoL, concluded that patients with a lower level of Karnofsky performance status <80%, which means performing normal activities with difficulty, show a worse body image (*p* = 0.026) [15].

Quality of life (QoL), and especially health-related quality of life (HRQoL) is a multidimensional concept relating to the areas of physical fitness, mental wellbeing, emotions, spirituality, and social functioning. Quality of life (QoL) has been defined by the WHO as “an individual’s perception of their position in life in the context of the culture and value systems in which they live and in relation to their goals, expectations, standards and concerns” [16]. More and more important in the treatment process, apart from the physiological assessment, is the assessment of quality of life (QoL), used in the assessment of the health outcomes of therapeutic programs. A high QoL additionally improves communication with the patient and his or her involvement in the treatment process. QoL is of great importance in the case of diseases that are difficult to treat, such as cancers, especially those with a poor prognosis, such as pancreatic cancer [17]. WHOQOL-100 is a quality-of-life assessment developed by the WHOQOL Group with fifteen international field centres to develop a quality-of-life assessment that could be applied interculturally [17]. QoL areas and its components defined by WHO are presented in the Table 1 below. Those areas were used as guidance on data extraction serving as a QoL framework.

Health and ongoing therapy have a direct impact on the first four areas defining quality of life. In the case of the environment and personal values and beliefs, the impact on health is indirect. HRQoL focuses on the impact of health on the patient’s quality of life. HRQoL is used in medicine to measure the effects of chronic diseases, therapy, and short-term and long-term disability. A high index value of HRQoL indicates that despite the disease, the patient is perceived as functioning well. On the other hand, a low level of HRQoL illustrates the limitations experienced by the patient [18].

Due to the wide scope of the concept of quality of life, many tools have been developed to measure it. Most often these are questionnaires addressed to the patient [18,19]. The SF-36 questionnaire, its shortened version SF-12, and EQ-5D are commonly used to assess the quality of life, allowing for comparisons with the general population or with a control group without cancer. In oncology, the functional assessment of cancer therapy-general (FACT-G), the European Organization for the Research and Treatment of Cancer QLQ-C30 (EORTC QLQ-C30), and the QoL-CS are often used. The most widely used EORTC QLQ-C30 tool consists of 30 components. The questionnaire is used in prospective clinical trials and it consists of five functional assessment scales (roles, physicality, emotions, cognitive abilities, and social functioning), three scales measuring symptoms (pain, nausea, vomiting, and fatigue), the global health scale, and quality of life, additional symptoms reported by cancer patients (dyspnoea, loss of appetite, sleep disturbances, constipation, diarrhea), and the perception of the impact of the disease and treatment on the patient’s economic situation [20]. The results are measured on a scale from 0 to 100, a higher score indicates better functioning during the treatment of the disease and a less negative impact of the disease symptoms.

The evaluation of the quality of life has become an important part of clinical trials in oncology, especially in the third phase of research. The magnitude of the clinical benefit scale, developed by the European Society of Medical Oncology (ESMO) also includes quality of life as an important parameter [21]. The American Society of Clinical Oncology (ASCO), in its recommendations for the evaluation of the results of clinical trials of cancer drugs, specified quality of life as an assessment index [22,23].

Due to late detection, low survival rate, and an increasing number of new cases of this cancer, it is important to pay attention to the quality of life of patients. According to research, people suffering from pancreatic cancer often have a lower quality of life than other cancer patients. Due to this phenomenon, it is important to increase knowledge of this subject and one solution is a literature review presenting new, relevant knowledge.

The objective of the study was to present the current state of knowledge on the quality of life of patients diagnosed with pancreatic cancer, factors determining QoL, and ways of coping with the disease.

## 2. Materials and Methods

In the period of July–August 2022, a literature review was carried out to summarize the available knowledge on the quality of life of patients with pancreatic cancer, including pain and depression as important factors in measuring the quality of life and cognitive function of patients. The search strategy and Boolean logical operators were used as follows: “pancreatic cancer” or “pancreatic ductal adenocarcinoma” and “quality of life” and “pain management” or “pain” and “mental adjustment to disease” and “depression”. Literature searches were conducted in Pubmed and Cochrane. The search terms included both MeSH terms and text words. A search for grey literature was also made. The search was augmented by a hand search of relevant articles included in the literature review. On the basis of the above strategy, the most important literature items in this field were identified using the authors’ expertise on the subject of cancer and the quality of life associated with the disease. The publication inclusion criteria according to the PICOS scheme are presented in the Table 2 below.

**Figure 1:**
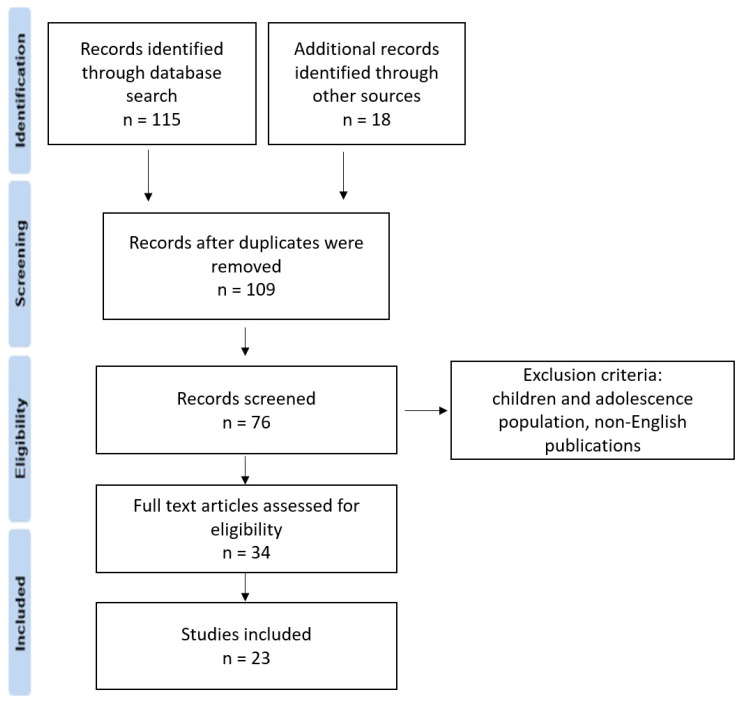
Simplified PRISMA scheme.

The search was limited to publications in English. Observational studies and case studies were also included due to their value in the topic of depression and cognitive abilities among patients with pancreatic cancer. Exclusion criteria included a population under the age of 18. Initially, two authors read the titles and abstracts of all searched publications, and clearly irrelevant studies were excluded. Subsequently, all potentially eligible articles were subjected to a thorough independent evaluation by two other authors to determine their eligibility. Disagreements were resolved by consensus with the participation of a third independent analyst. The simplified PRISMA scheme is presented to clarify the selection process (Figure 1). Finally, twenty-three articles were recognized by all analysts as eligible for the review.

## 3. Results

### 3.1. Location of Tumour and Survival Rates

Among patients with pancreatic cancer, a decrease in the quality of life was observed due to the severe course of the disease, the burden of physical symptoms, and the side effects of therapy [24,25]. The symptoms affecting the quality of life observed in patients depend, among others, on the clinical image of the cancer—the location of the cancer and its stage. In the case of cancer located on the head of the gland, obstructive jaundice develops in 70–80% of cases. Duodenal infiltration with subsequent high obstruction of the gastrointestinal tract may also occur [26]. However, it has been shown that the primary location of cancer in the head of the pancreas at the time of diagnosis is a predictor of better survival [26,27]. Cancer located in the body or tail of the pancreas is usually larger, more often causes pain (e.g., under the scapula) and metastases, and is more often unresectable compared to cancer located in the head of the pancreas, which negatively affects QoL [28]. The quality of life depends on the treatment.

### 3.2. QoL with Pancreatic Cancer

Studies have shown that HRQoL may temporarily decrease after resection and may improve after a full cycle of chemotherapy [9,29]. In terms of chemotherapy, the type of therapy is important. In palliative treatment, 5-fluorouracil (5-FU) monotherapy has been the standard of care in metastatic cancer for years, providing a 10% objective response rate, however, it has not shown a positive effect on QoL. Therapy using 5-FU was replaced in 1997 with gemcitabine, which resulted in better pain control in every fourth metastatic patient and improved QoL. Palliative therapies in which gemcitabine was combined with another cytostatic (e.g., capecitabine, cisplastin) did not improve the quality of life. Multidrug palliative chemotherapy with the FOLFIRINOX regimen (5-fluorouracil biomodulated with folinic acid, irinotecan, and oxaliplantin), introduced in 2011, was characterized by higher toxicity than gemcitabine in the degree ≥G3, i.e., neuropathy (46% vs. 21%, *p* < 0.001), thrombocytopenia (9% vs. 4%, *p* = 0.04) and diarrhoea (9% vs. 4%, *p* = 0.04) were more common. Despite the higher toxicity of the FOLFIRINOX regimen, multidrug chemotherapy had a positive impact on the quality of life of patients, reducing the relative risk of QoL deterioration by 63% (HR 0.47; 95% CI: 0.3–0.7). In 66% of patients receiving gemcitabine therapy, QoL worsened after six months compared to 31% of patients treated with FOLFIRINOX [29]. Among patients after resection of peripapillary adenocarcinoma, multivariate analysis, including prognostic variables, showed a statistically significant survival benefit associated with adjuvant chemotherapy [30]. On the other hand, the APACT study with 866 patients showed benefits in terms of overall survival with nab-paclitaxel and gemcitabine used in the adjuvant therapy of patients after resection of pancreatic cancer [31]. In the case of therapy with nab-paclitaxel and gemcitabine, the median overall survival was 41.8 months compared with 37.7 months for gemcitabine alone (HR = 0.80; *p* = 0.0091). The five-year overall survival rate was 38% for combination therapy vs. 31% for gemcitabine [31]. Combination therapy might be beneficial for patients who are ineligible for FOLFIRINOX therapy.

Gemcitabine-based therapies constitute the standard of care for patients with locally advanced or metastatic pancreatic ductal adenocarcinoma [32]. In 2013, a study conducted by Von Hoff et al. was published, which examined the safety and efficacy profile of nab-paclitaxel therapy in combination with gemcitabine compared to gemcitabine monotherapy in 861 patients with metastatic pancreatic cancer [33]. Treatment with nab-paclitaxel in combination with gemcitabine has been shown to significantly improve overall survival, progression-free survival, and response rate. However, combination therapy was associated with an increase in the incidence of peripheral neuropathy and myelosuppression. In a study conducted by Wang-Gillam et al. in 2016 on 417 patients previously treated with gemcitabine-based therapy, the effect of monotherapy with nano-liposome irinotecan was assessed in comparison to combination therapy with fluorouracil and folinic acid in metastatic pancreatic cancer (NAPOLI-1 study) [34]. Safety level was assessed in all patients who had received study therapy. Combination therapy has been shown to prolong survival among patients previously treated with gemcitabine. It has also been proven that there is no deterioration in the quality of life when using combination therapy.

Pancreatic adenocarcinoma is one of the cancers with the worst prognosis. It is an aggressive disease, and the burden of physical symptoms, combined with the undesirable effects of chemotherapy, leads to a deterioration of the patient’s quality of life [35]. In metastatic pancreatic cancer, chemotherapy, according to the FOLFIRINOX regimen and nab-paclitaxel + gemcitabine, has shown significant survival benefits. However, due to the aggressive course of the disease, the quality of life of these patients drops sharply. In a study by Gourgou-Bourgade et al., the effect of FOLFIRINOX therapy (oxaliplatin, irinotecan, fluorouracil, and leucovorin) was evaluated in comparison with gemcitabine on the quality of life of patients with metastatic pancreatic cancer [36]. Improvement in general health (GHS; *p* < 0.001) was demonstrated in the FOLFIRINOX group, and improvement in emotional functioning (*p* < 0.001) was demonstrated in both groups, along with the reduction in pain, insomnia, anorexia, and constipation in both groups. A significant increase in diarrhoea was observed in the FOLFIRINOX group during the first two months of chemotherapy. Time until ultimate deterioration was significantly longer for the FOLFIRINOX group compared to the gemcitabine group with regards to GHS, physical functioning, cognitive and social function, and six symptom domains (fatigue, nausea/vomiting, pain, dyspnoea, anorexia, and constipation). Physical functioning, constipation, and dyspnoea were independent significant predictors for survival in the treatment group, age over 65, and low serum albumin levels [36]. In a study conducted by Al-Batran et al. quality of life was assessed using a self-completed questionnaire, validated standardized data collection forms from the European Organization for Research and Treatment of Cancer EORTC QLQ-C30 questionnaire for quality of life [37]. Quality of life was assessed at the beginning of the study, every four weeks on day one of each cycle for six months, and four weeks after the last cycle in the study. The primary endpoint was the proportion of patients who did not experience any deterioration (i.e., improvement or stability). A 5% change was considered clinically significant. Change in the quality of life over time was estimated using the Kaplan-Meier (KM) method, and 61% of patients did not observe any deterioration after three months. Maintaining an adequate quality of life in pancreatic cancer patients seemed to be achievable in the subgroup of patients receiving first-line combination chemotherapy [37].

### 3.3. Pain Associated with the Disease

In advanced and metastatic pancreatic cancer, pain is a common problem for the patient. From the point of view of pathophysiology, pain can be divided into visceral, somatic, or neuropathic. Pain in pancreatic cancer may be caused by the cancer itself (infiltration of the nerve plexus and nerve branches, spinal metastases, peritoneal dissemination with subocclusion, etc.) or resulting from the applied treatment (surgery, radiation therapy, and chemotherapy) [35]. Chronic pain resulting from advanced pancreatic cancer is reported by over 50% of patients. Treating and managing pain is a key issue in quality of life. There has been considerable discussion on the positive effect of pain control on increasing patient survival. Individualized pharmacotherapy is considered the mainstay of treatment for pain control in cancer patients [35]. Research indicates that inadequate pain management is associated with eating disorders (reduced caloric intake), sleep disorders, and reduced professional and social life. Inadequately treated pain can affect the patient’s tolerance to chemotherapy. Patients with a lower level of pain have a higher quality of life [38]. In the treatment of chronic cancer pain, oral morphine is the basic strong classic opioid analgesic, next to the transdermal form of fentanyl and oral oxycodone and the partial antagonist of the opioid receptors—transdermal μ—buprenorphine—which works better in neuropathic pain. Most often, classic opioids are administered in combination with coanalgesics with a supporting effect. In some cases, other pain management procedures are used, such as radiation therapy, which is effective in controlling pain resulting from the compression of a large tumour on other organs or structures. Radiation therapy reduces the tumour and the pressure it causes. In contrast to the rapid action of analgesics, the effect of radiation therapy is noticeable only a few weeks after radiation [38]. Good analgesic effects are also brought by a splanchnicectomy—surgical, laparoscopic removal of the ventral sympathetic ganglia. The systematic review by Erosa et al. presents available studies using the atypical opioids tapentadol, buprenorphine, and levophanol in the treatment of neuropathic pain [39]. A total of ten out of 1619 studies were included in the review. Of the five tapentadol studies included, four studies showed that tapentadol monotherapy is effective in the treatment of diabetic peripheral neuropathy or chronic radiating low back pain. Of the three studies included in the buprenorphine study, only one was a randomized controlled trial that found no statistically significant reduction in pain with buprenorphine TD, possibly due to the very high withdrawal rate during the study. Only two case reports on levorphanol are included in the available literature and they provide low-quality anecdotal evidence. Therefore, more research, especially RCT, to evaluate the true efficacy and risks of each agent is needed. The Fan and Zhou study, on the other hand, evaluated the short-term therapeutic effect of computed tomography-guided radioactive 125I seed implantation (CTRISI) in the treatment of pain in patients with advanced pancreatic cancer [40]. Thirty-seven patients with advanced cancer were included in the study. The level of cancer pain and the daily dose of hydroxycodone were compared before and after CTRISI using a numerical rating scale (NRS). It was shown that CTRISI can effectively relieve pain in patients and inhibit local tumour progression. This technique helps relieve pain symptoms, reduce the amount of painkillers used, and improve the QoL of patients [40].

### 3.4. Coping Strategies

Apart from medical factors, the quality of life of patients is also influenced by their internal resources and the adopted strategy for coping with the disease. Among internal strategies for coping with cancer pain, the strategy of praying/faith showed the best results based on the BPCQ, while in the area of mental adaptation to the disease, positive revaluation in patients with pancreatic cancer achieved the highest levels of the index when assessed using the Mini-Mac tool, which is consistent with the study conducted by Religioni et al. [41]. Some studies have suggested a link between the intensification/onset of depression and pancreatic cancer. Oncological patients are more prone to depression. The results of the studies conducted by Burke et al. indicate that depression develops in 15 to 30% of cancer patients, with exposure three to five times higher than in the general population [42]. In addition to the psychological response to the diagnosis, some studies indicate a relationship between depression and the disease process itself, specifically with biomarkers of inflammation in the blood (cytokines, including interleukin-6, which is a signalling particle responsible, among others, for initiation and development of the inflammatory response, induction of acute phase protein synthesis, or activation and differentiation of T lymphocytes). Cytokines, including IL-6, may play the role of mediators of the depressive reaction, probably by their influence on the metabolism of neurotransmitters and their neuroendocrine function [43]. People with pancreatic cancer tend to have poorer QoL scores in the field of mental health in comparison to other cancers. In a study conducted by Clark et al., patients with pancreatic cancer often reported symptoms of mental anxiety, depression, or somatization of stress in comparison with other cancer patients [44]. Psychosocial factors, poor prognosis, long and burdensome treatment, and accompanying dysregulation of the immune and endocrine systems are likely causes of mental imbalance [45]. The inclusion of mental health professionals in the medical team is one of the recommendations of the American College of Surgeons and the Institute of Medicine for routine screening in clinical oncology [46]. Another psychological factor that has an impact on the quality of life is the fear of the recurrence of the disease [47]. In study results based on 188 patients who underwent potentially curable surgery of pancreatic ductal adenocarcinoma, peripapillary adenocarcinoma (adenocarcinoma papillae Vateri), or pancreatic neuroendocrine tumours, the fear of recurrence of cancer was over 50% [47]. Also, social support has an impact on the quality of life of patients with pancreatic cancer. According to the Lee J. social support study, it has an indirect impact on the quality of life and a direct impact on the perceived health status and functional status [48]. This suggests that while social support does not directly affect QoL, it acts as a QoL mediator that modulates the effects of functional status and perceived health. Patients receiving high levels of social support have social skills to some extent, and higher levels of support are associated with fewer emotional symptoms, such as depression and anxiety, and reduced physical symptoms. In addition, social support is associated with improved prohealth behaviours and a higher quality of life. Of the entire social support system, family support had the highest scores and was of the highest importance to patients [48].

### 3.5. Cognitive Functions in Patients with PC

Cognitive decline has also been observed in patients with pancreatic cancer [49,50,51]. Research also shows that depression in patients with PC may result from biological changes that are caused by the presence of the tumour itself [51]. Patients with pancreatic cancer who suffered from depression showed greater cognitive difficulties compared to patients suffering from depression and other cancers [52].

#### Limitations

This article is not a systematic review. The advantage of the article is taking into account the latest available publications on the quality of life in cancer patients, internal resources and strategies for coping with the disease adopted by patients, and changes in cognitive functions accompanying the disease. So far, no study has been published on this subject in terms of the quality of life with pancreatic cancer, especially considering cognitive and mental aspects.

## 4. Conclusions

The low curability and low survival rate of pancreatic cancer significantly affect the quality of life of patients, often in the form of its significant deterioration, especially in terms of mental changes and cognitive functions as well as coping with cancer. Somatic disorders are easier to treat with appropriate therapeutic procedures, which translates into an improvement in the quality of life in this area. In order to properly monitor the quality of life of patients, it is necessary to use standardized measurement tools, such as standardized questionnaires. Researchers should also expand the catalogue of available psychometric tools in order to monitor the patient’s response to the disease and therapy as closely as possible. Due to the proven occurrence of depression among patients with pancreatic cancer, psychological and psychiatric support should be included in the treatment process. Cognitive decline with comorbid depression is also typical for patients with this type of cancer. Attention should also be paid to the therapy of fear of recurrence of the disease, which accompanies patients even after surgical procedures that give the possibility of remission. According to scientific evidence, patients who have greater internal resources and support have better-developed mechanisms for coping with the disease and cope better with therapy, which is particularly important in pancreatic cancer. The low quality of life associated with health, especially mental and social, among patients with pancreatic cancer requires further research, the results of which should improve the situation of patients in this area.

This study can be used as a source of knowledge for practitioners taking part in the healthcare of patients with pancreatic cancer. Due to high mortality and late diagnosis, it is important to ensure a high quality of life for patients. A higher level of QoL affects better coping with the treatment process and ailments, especially pain, related to the disease.

## Figures and Tables

**Table 1:** Quality-of-life areas as defined by WHO based on WHQOL-100.

Quality of Life Area	Component Areas of Quality of Life
Physical health (HRQoL)	energy and fatigue, pain and discomfort, sleep, and rest
Mental health (HRQoL)	perception of your body and appearance, negative feelings, positive feelings, self-esteem, thinking, learning, memory, and concentration
Independence level (HRQoL)	mobility, activity related to everyday life, dependence on drugs and medical products, work efficiency
Social relations (HRQoL)	interpersonal relations, social support, and sexual activity
Environment	economic situation, sense of freedom and security, health and social care—availability and quality, home environment, rest, and spending free time, transport
Personal values and beliefs	religion, faith, personal beliefs

**Table 2:** Criteria for publication inclusion according to the PICOS scheme.

Population (P)	Patients with Pancreatic Cancer
Intervention (I)	Any or none
Comparator (C)	Any or none
Effects (O)	Quality of life of patients with pancreatic cancer, coping strategies, depression, and pain management
Type of studies (S)	RCT, Case studies, prospective studies, systematic review,
Study period	2010 onwards
Limitations	Publications in English assessing the impact of pancreatic cancer on the quality of life
Exclusion	Patients aged < 18 years, studies before 2010, and non-English publications

## Data Availability

Not applicable.
